# Cyclocurcumin as Promising Bioactive Natural Compound: An Overview

**DOI:** 10.3390/molecules29071451

**Published:** 2024-03-24

**Authors:** Carla Gasbarri, Guido Angelini

**Affiliations:** Department of Pharmacy, University “G. d’Annunzio” of Chieti—Pescara, Via dei Vestini, 66100 Chieti, Italy; guido.angelini@unich.it

**Keywords:** cyclocurcumin, docking analysis, biological activity, platelet aggregation, rheumatoid arthritis, fluorescence, photoisomerization, solvatochromism, bioavailability

## Abstract

Although identical in molecular formula and weight, curcumin and cyclocurcumin show remarkable differences in their reactivity. Both are natural compounds isolated from the rhizome of turmeric, the former is involved in the diketo/keto-enol tautomerism through the bis-α,β-unsaturated diketone unit according to the polarity of the solvent, while the latter could react by *trans*-*cis* isomerization due to the presence of the α,β-unsaturated dihydropyranone moiety. Even if curcumin is generally considered responsible of the therapeutical properties of *Curcuma longa* L. due to its high content, cyclocurcumin has attracted great interest over the last several decades for its individual behavior and specific features as a bioactive compound. Cyclocurcumin has a hydrophobic nature characterized by fluorescence emission, solvatochromism, and the tendency to form spherical fluorescent aggregates in aqueous solution. Molecular docking analysis reveals the potentiality of cyclocurcumin as antioxidant, enzyme inhibitor, and antiviral agent. Promising biological activities are observed especially in the treatment of degenerative and cardiovascular diseases. Despite the versatility emerging from the data reported herein, the use of cyclocurcumin seems to remain limited in clinical applications mainly because of its low solubility and bioavailability.

## 1. Introduction

*Curcuma longa* L. is a perennial herbaceous plant of the Zingiberaceae family commonly referred as turmeric and has been extensively applied for centuries in the traditional medicine of Asian countries as panacea for a series of diseases, including allergy, depression problems and cardiovascular disorders [1,2,3,4,5,6]. Modern studies have attributed the anti-inflammatory, neuroprotective, antioxidant, hepatoprotective and antidiabetic properties of turmeric to curcuminoids, a class of polyphenols isolated from the rhizome of the plant, also responsible for the typical yellow color of the curry spice and often added as colorant in food [7,8,9,10,11,12,13,14].

Although more than 50 natural curcuminoids have been identified till now, curcumin represents the major component of turmeric, followed by demethoxycurcumin and bisdemethoxycurcumin [15,16].

Curcumin, demethoxycurcumin and bisdemethoxycurcumin possess a bis-α,β-unsaturated diketone moiety in their structure and exhibit diketo/keto-enol tautomerism. Generally, the intramolecularly hydrogen-bonded keto-enol is the prevalent form in solution, and the absorption band in the range 408−434 nm due to the π-π* transition can be observed in the UV–vis spectra [17,18]. The therapeutical properties of turmeric are mainly attributed to curcumin due to its high concentration (75–80%) in comparison to demethoxycurcumin (15–20%) and bisdemethoxycurcumin (3–5%) [19]. Moreover, some studies revealed that most of the biological effects of curcumin may be dependent on the concentration of enol [20,21].

**Figure 1:**
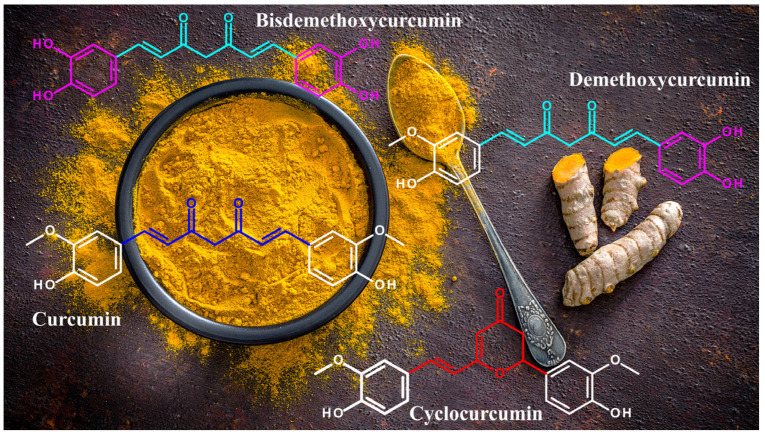
Structures of curcumin, demethoxycurcumin, bisdemethoxycurcumin and cyclocurcumin from turmeric.

**Scheme 1:**
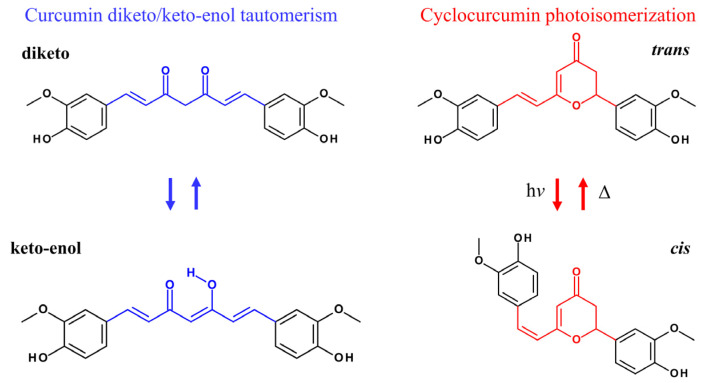
Comparison between the diketo/keto-enol tautomerism of curcumin (on the **left**) and the photoisomerization of cyclocurcumin (on the **right**).

The individual contribution of curcuminoids other than curcumin to the remarkable properties of *Curcuma longa* L. was tested, showing for each compound antiproliferative effects and efficacy against neurodegenerative and vascular diseases. Interestingly, synergistic activity was observed in the case of mixture and association with curcumin [22,23,24]. The presence of the diketone moiety seems to be directly related to the inhibition of tumor-cell proliferation induced by curcuminoids, whereas minor activity was observed for the minor ingredient isolated in turmeric identified as cyclocurcumin (CyCur) [25,26]. Although identical molecular formula (C_21_H_20_O_6_) and molecular weight (368.38), CyCur differs significatively from curcumin in structure and reactivity: the molecule appears as a non-diarylheptanoid curcuminoid in which the diketone moiety is replaced with an α,β-unsaturated dihydropyranone unit (Figure 1). For this reason, the diketo/keto-enol tautomerism observed for curcumin, demethoxycurcumin, and bisdemethoxycurcumin is excluded in the case of CyCur and replaced by isomerization in which rotation around the ethylenic double bond occurs under exposition to light [27] (Scheme 1).

In recent years cyclocurcumin attracted great attention as a natural bioactive compound despite the low concentration in nature and the low solubility in aqueous solution. In this review, manifold aspects of chemical and biological behavior of CyCur beside docking analysis are reported for a better comprehension of its properties for the development of novel formulations based on curcuminoids.

## 2. Structural and Chemical Properties

The bioactive phytocompound from turmeric with the IUPAC name 2-(4-hydroxy-3-methoxyphenyl)-6-[(*E*)-2-(4-hydroxy-3-methoxyphenyl)ethenyl]-3,4-dihydro-2*H*-pyran-4-one and commonly referred as cyclocurcumin (CyCur) was firstly isolated and identified in 1993 by Kiuchi et al. in the valuation of the nematocidal activity of curcuminoids [28]. The presence of CyCur at low yield (0.8%) in the chloroform extract after silica gel chromatography was initially attributed to the rearrangement of curcumin through intramolecular Michael addition involving the enol oxygen and the enone group. The identification of CyCur as an individual compound was then demonstrated by HPLC, ^1^H-NMR, ^13^C-NMR, HRMS and UV–vis analysis.

An increase in the yield (21%) was obtained after chromatographic purification by performing the synthesis of CyCur, starting with curcumin in trifluoroacetic acid. The cyclization reaction was carried out in dry benzene at room temperature for 65 h in the dark to avoid the photoisomerization of the product. The synthesis was modified later by Randino and coworkers by using microwave at 100 °C for 4 min with trifluoroacetic acid 100% in solvent-free condition to reach a yield of 10% [29]. Pure CyCur appears as a yellow powder having a melting point between 179 and 226 °C, a boiling point at 571.9 ± 50 °C at 760 mmHg and a density of 1.4 ± 0.1 g/cm^3^ [30].

**Figure 2:**
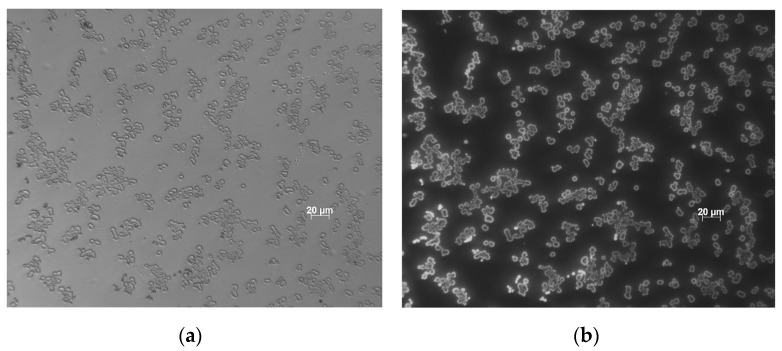
Optical micrographs of CyCur aggregates in aqueous solution under visible light (**a**) and in fluorescence (**b**). Data from reference [33].

Generally, ethanol and concentrated acetic acid are employed as solvents by considering the high hydrophobic nature of the compound [31]. The cLogP value of 3.34 and the LogS of −3.47 were calculated by in silico studies in which the drug-likeness prediction was tested to evaluate the absence of tumorigenicity, mutagenicity, irritation and reproductive toxicity risk in the use of CyCur as a potential drug candidate [32]. The lipophilic character of the compound is also confirmed by its tendency to self-assemble in aqueous solution to form spherical and monodispersed aggregates having nanometric size. This behavior was microscopically demonstrated under visible light and in fluorescence [33]. The optical micrographs of CyCur aggregates observed in aqueous solution are reported in Figure 2.

**Figure 3:**
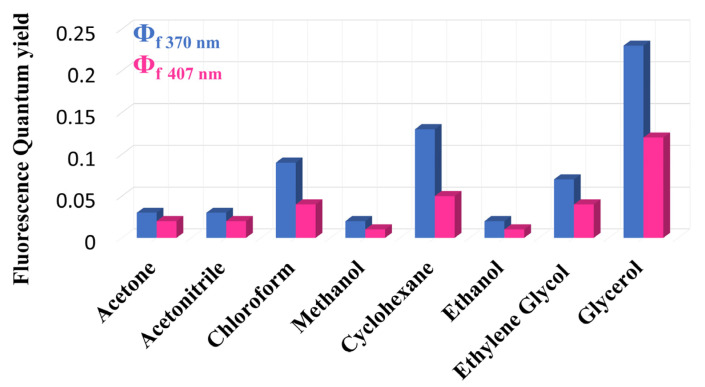
Fluorescence quantum yield (ϕ_f_) of CyCur calculated, exciting at 370 nm (blue color) and at 407 nm (magenta color) in organic solvents reported in order of increasing viscosity at 25 °C. Data from reference [27].

The photophysical properties of CyCur depends on the surrounding environment as demonstrated by Adhikary et al. [27]. It was observed that the position of the maximum in the fluorescence spectrum depends both on the excitation wavelength and the solvent polarity. Fluorescence quantum yield of the molecule (ϕ_f_) tends to increase significantly with the solvent viscosity at 25 °C. The ϕ_f_ values calculated for cyclocurcumin solution in protic and aprotic solvents by using 370 and 407 nm as excitation wavelength are reported in Figure 3.

**Figure 4:**
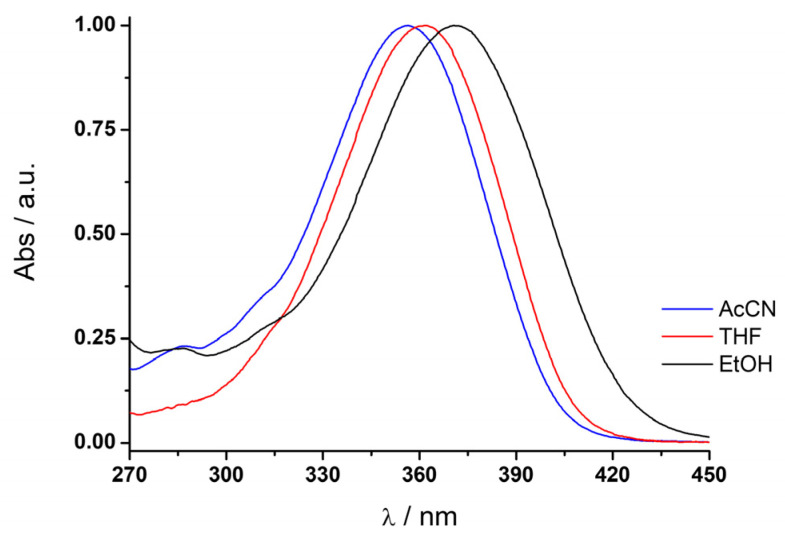
Normalized UV–vis spectra in acetonitrile (AcCN), tetrahydrofuran (THF) and ethanol (EtOH) determined at 25 °C as examples of the solvatochromism of CyCur. Data from reference [36].

Time-resolved studies suggested nonexponential fluorescence decay in the investigated protic and aprotic solvents according to the generation of rotational isomers of cyclocurcumin due to *trans*-*cis* isomerization. The *trans* isomer is about 30 kcal/mol more stable in comparison to the *cis* form and represents the dominant state of the molecule both in turmeric and in solution. Generally, the spectral behavior of cyclocurcumin may change according to the solvent properties. In particular, the Catalán and Spange empirical solvent parameters scale applied to the solvatochromism of cyclocurcumin revealed that polarizability plays athe major role in the solute–solvent interactions in comparison to dipolarity and acid–base interactions [34,35]. The normalized UV–vis spectra of cyclocurcumin in acetonitrile (AcCN), tetrahydrofuran (THF) and ethanol (EtOH) are shown in Figure 4 as an example of the solvatochromism of the molecule.

Photoisomerization of cyclocurcumin can be induced under UV or visible light, at 365 and 436 nm, respectively. Interestingly, irreversible thermal degradation of cyclocurcumin into curcumin over time was also spectroscopically observed [36]. The conversion of the CyCur *trans* isomer into the *cis* form is confirmed by the comparison of the spectral behavior of the molecule before and after irradiation: the HPLC analysis of ethanolic solution of cyclocurcumin, for example, shows a dynamic chromatogram in which two resolvable stereoisomers interconverting on the separation time scale can be detected; moreover, the decreasing of the maximum absorption emission band centered at 370 nm and the simultaneous increasing of the weak band at 286 nm can be observed in the UV–vis spectrum. The interconversion between the *trans* and *cis* isomers can be demonstrated by ^1^H-NMR analysis: the reduction in the homonuclear ^3^J ethylenic ^1^H coupling from 16 Hz to 12.8 Hz, the reduction in the signals from 3.812/3.808 ppm associated with the *trans* isomer, and the appearance of the signals from 3.678/3.773 ppm associated with the *cis* isomer can all be observed in DMSO-d6. The chemical shifts of the protons determined in the spectra of the isomers of CyCur in DMSO-d6 are reported in Table 1.

The thermal *cis*-*trans* isomerization of cyclocurcumin takes place in the dark according to a first-order kinetic profile. The first-order kinetic rate constants (*k*_obs_) were measured at different temperatures in organic solvents and in various chemical environments, including silver nanoparticle aqueous solution. The comparison of the *k*_obs_ values determined in the temperature range 294–314 K in ethanol, pure water and in silver nanoparticle aqueous solution suggests that the negatively charged nanoparticles might favor the isomerization of the *cis* form of CyCur into the *trans* isomer as previously observed in the case of azobenzene derivatives in aqueous solution [37,38].

## 3. Computational Studies

Recently, the antioxidant properties of cyclocurcumin against free radicals were theoretically investigated by Li et al. in water and chlorobenzene [39]. The bond dissociation enthalpy (BDE), the ionization potential (IP), the proton dissociation enthalpy (PDE), the proton affinity (PA) and the electron transfer enthalpy (ETE) were calculated for the molecule in the neutral and deprotonated state. The thermodynamic parameters were obtained for the three most common radical scavenging mechanisms: HAT, SET-PT and SPLET. The Hydrogen Atom Transfer (HAT) consists of a single-step reaction by which the homolytic cleavage of the hydroxyl bond generates stable radicals; the Single-Electron Transfer-Proton Transfer (SET-PT) is a two-step reaction in which the formation of a radical cation from the neutral antioxidant is followed by the generation of a radical; the Sequential Proton Loss Electron Transfer (SPLET) is a two-step reaction in which the formation of an anion from the neutral antioxidant is followed by the generation of a radical. The reaction and activation free energies between the phenolic hydroxyls of CyCur and free radicals ^•^OH and ^•^OOH were also calculated for each mechanism. The data indicated that HAT was the most probable scavenging mechanism for cyclocurcumin in neutral and deprotonated form. Stronger activity against ^•^OH in comparison to ^•^OOH was observed in the investigated conditions.

The potentiality of cyclocurcumin in the modulation of oxidative process was demonstrated by in silico studies focused on the systemic inflammation induced by C-reactive protein [40]. Efficacy against the photoaging process based on the tryptase activity was also determined. In this case, a strong inhibition of the enzyme occurs due to the ability of the CyCur molecule to bind the tryptase monomer in all the possible sites [41].

Molecular docking studies identified cyclocurcumin as a potential lead compound for the development of dual inhibitors of DNA topoisomerases I and II [42]. Among the docked curcumin derivatives, the lowest free energy bindings were observed in the case of CyCur for topoisomerase I (ΔG value −10.33 kcal/mol) and for topoisomerase II (ΔG value −11.16 kcal/mol). The essential role of these enzymes in repairing DNA from alteration damages is well known, and the docked data indicated the binding of CyCur at the DNA cleavage site by forming complexes with topoisomerases I and II, which are converted into permanent DNA strand breaks by cellular processes inducing apoptosis. Furthermore, computational data on the inhibition of *Streptococcus mutans* deoxycytidylate deaminases highlighted the potentiality of the CyCur molecule in the formulation of organic mouthwash acting as the anti-biofilm drug for the prevention of the dental caries [32].

Recently, Singh and Misra studied the inhibition of Influenza A virus induced by marketed drugs, curcuminoids, including cyclocurcumin and two curcumin derivatives [43]. They reproduced the sequence of polymerase PB2 identified in the virus of the swine flu to develop a protein for docking analysis [44,45]. The comparison of the calculated parameters based on run, minimum binding energy and number of intramolecular and intermolecular hydrogen bonds revealed that cyclocurcumin represents the most favored among the investigated compounds for the inhibition of the polymerase PB2.

Bioinformatic analysis focused on the antiviral activity of curcuminoids against SARS-CoV-2 was also carried out [46]. The inhibition of the viral replication was demonstrated and attributed to the affinity of the investigated ligands to the non-structural protein 3 (NSP3) expressed as low binding energy. The lowest values based on rerank score was calculated for CyCur (−128.38 kcal/mol) in comparison to curcumin (−114.270 kcal/mol), demethoxycurcumin (−111.967 kcal/mol) and bisdemethoxycurcumin (−106.879 kcal/mol). Interestingly, the result reported for the CyCur molecule is very close to the value obtained for ribavirin (−127.920 kcal/mol).

Another recent study of molecular docking simulation compared the binding affinity with spike protein of SARS-CoV-2 of some components of Indian spices in their free form and as hyaluronic acid (HA) conjugates. The density functional theory data indicated for the HA-CyCur conjugate the greatest binding affinity according to the lower energy gap of −0.0763, 0.1426 high electron negativity and 0.0763 electron affinity [47]. Furthermore, the role of cyclocurcumin as a non-nucleotide anti-polymerase agent in the development of potential inhibitors against the RNA-dependent RNA polymerase and its affinity for the active site of SARS-CoV-2 main protease was demonstrated [48,49].

Molecular modeling calculations in association with electron paramagnetic resonance and circular dichroism studies were performed by Randino et al. to determine the specific role of the curcuminoids as neuroprotective agents in preventing the formation of the β-amyloid fibrillar aggregates commonly observed in the neurodegenerative diseases [29,50,51,52,53]. The individual interaction of cyclocurcumin with the β-amyloid fragment Aβ (25–35) was investigated in hexafluoro-2-propanol/water mixtures and liposomal solutions [54]. Small unilamellar vesicles composed of pure zwitterionic 1,2-dioleoyl-*sn*-glycero-3-phosphocholine (DOPC), pure anionic 1,2-dioleoyl-*sn*-glycero-3-phospho-*rac*-1-glycerol (DOPG) and DOPC/DOPG in a 90/10 molar ratio mixture were prepared by hydration of the phospholipidic film with phosphate buffer at pH 7.4, followed by sonication. Liposomes and vesicles are commonly used as model membranes, drug delivery systems and membrane mimicking environments for specific reactions; in this case, the DOPC, DOPG and DOPC/DOPG liposomal solutions allowed to thoroughly investigate the role of the tested curcuminoids on Aβ (25–35) and the effects on membrane perturbation [55,56,57,58]. Unlike the other curcuminoids positioned in the proximity of the membrane, the cyclocurcumin molecule penetrates the phospholipid bilayer and directly interacts with Aβ (25–35). Interestingly, the data obtained in the hexafluoro-2-propanol/water solution and in DOPG liposomes indicate that CyCur promotes a remarkable alteration of the Aβ (25–35) conformational equilibrium by reproducing the pattern of the α-helical coiled-coil structures involved in the binding of the peptides with the biological membrane [59]. This behavior is due to the *semi-folded* conformation in which cyclocurcumin is structurally constrained from an intramolecular cyclization and leads to the protection of the cellular membrane by hampering the formation of fibrillar aggregates based on α-helical coiled-coil structures [60,61]. Interestingly, four subfamilies of conformers were obtained by molecular mechanism optimization (MM) and semi-empirical method (PM7): they show values of ΔE from the global energy minimum in the range 0.00–4.42 kcal/mol, different torsional angles and interatomic distances between the centroids of the two aromatic rings, from 7.00 to 9.66 Å, which referred to the lowest energy conformers of the family.

The neuroprotective effect of cyclocurcumin was confirmed through a cellular model based on neurodegenerative conditions as described in the next section.

## 4. Biological Activities

Chakraborty and coworkers [62] compared cyclocurcumin and curcumin properties as inhibitors of the neurotoxicity caused by 1-methyl-4-phenylpiridinium (MPP^+^) in neuronal-like differentiated PC12 cells from the pheochromocytoma of rat adrenal. The MPP^+^ selected as neurotoxin was able to approximate Parkinson’s disease conditions in the cell model [63,64]. The curcuminoids were tested against the cytotoxicity induced by MPP^+^ and intracellular reactive oxygen species (ROS) measurements., NADH fluorescence lifetime analysis [65,66,67] and cell viability studies were also performed.

**Figure 5:**
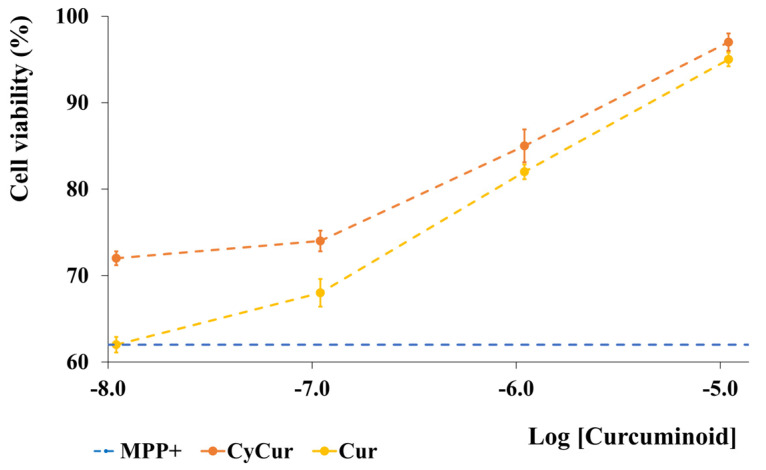
Cell viability test after the treatment with curcuminoids on the MPP^+^-pretreated PC12 cells. Statistical significance is in the range 0.001–0.05. Data from reference [62].

The results pointed out the lessening of the oxidative stress after curcumin and cyclocurcumin treatment. In both cases, the ROS level that increased by the neurotoxin was significatively reduced in the presence of curcuminoids, and the cellular metabolic activity was partially restored to normal conditions. The effects of curcumin and cyclocurcumin in the concentration range 0.01–10 μM on the cytotoxicity of PC12 cells pretreated with MPP^+^ were compared through the MTT-based cell viability test (Figure 5).

About 62% of cellular viability reduction was found by treating the selected cells with the neurotoxin at a concentration of 1 mM (blue line in the plot of Figure 5). A dose-dependent increase in the cell viability can be observed in the investigated conditions by adding CyCur and Cur (orange line and yellow line, respectively, in the plot of Figure 5). Furthermore, a higher protective activity of cyclocurcumin in comparison to curcumin can be detected in agreement with the overall data from the oxidative stress and fluorescence analyses.

Recently, Yang et al. identified CyCur as a promising natural compound for the treatment of rheumatoid arthritis by studying the interaction of a series of curcuminoids with the mitogen-activated protein kinase p38α, which is the protein directly involved in the regulation of the TNF-α expression [68]. Generally, a high level of tumor necrosis factor (TNF) can be observed in inflammatory disorders, so the over-expression of the TNF-α induced by p38α can be considered as an indicator for progressive autoimmune diseases as rheumatoid arthritis [69,70,71]. The studies pointed out that the immune-modulating role of cyclocurcumin in the inhibition of the TNF-α release is due to its affinity for the active site of p38α. The binding energy value of −6.12 kcal/mol and a maximum of five hydrogen bonds for the CyCur-p38α complex were obtained by molecular docking and molecular dynamic simulation analysis. Furthermore, the inhibitory activity of CyCur in the TNF-α release from lipopolysaccharide-stimulated human macrophages was determined by MTT assay. Interestingly, a similar dose-dependent effect was reported for cyclocurcumin and SB203580, a specific inhibitor of p38 mitogen-activated protein kinase.

The potential role of cyclocurcumin against cardiovascular disorders were highlighted by Chung and coworkers [72,73]. Inhibition effects on vasoconstriction and platelet aggregation were investigated. In the former case, the concentration-dependent activity of cyclocurcumin on the inhibition of phenylephrine-induced vasoconstriction in freshly isolated rat aortic ring system was observed, and the IC_50_ values of 14.9 (±1.0) μM was determined, showing the higher efficacy in comparison to curcumin (IC_50_ ˃ 100 μM). A concentration-dependent effect in the inhibition of L-type calcium channel-mediated vasoconstriction in primary vascular smooth muscle cells was also demonstrated. In the latter case, the role of CyCur in the treatment and prevention of thrombotic diseases was examined. A significant lessening in the shear stress-induced platelet aggregation/activation was observed and the IC_50_ values of 6.33 (±3.29) μM indicated a higher efficacy in comparison to curcumin (IC_50_ ˃ 250 μM). Interestingly, CyCur exhibits inhibitor activity against the platelet aggregation induced by different endogenous agonists as thrombin, collagen and ADP, without altering the blood clotting time or interfering with hemostasis [74,75,76].

## 5. Future Prospectives

Due to its high sensitivity to light and heat, low stability in aqueous solution and low bioavailability, the clinical applications of cyclocurcumin seem to be very limited [77]. In the case of curcumin, a series of strategies to improve the absorption and permeability avoiding the rapid metabolism and elimination was successfully proposed within the last few decades. Most of them are based on the incorporation or complexation of the molecule, involving supramolecular structures as liposomes, nanoparticles and cyclodextrins [78,79,80,81,82]. Generally, the curcumin-based formulations may be divided into three categories known as the first, second and third generation, for which a strong increase in the oral bioavailability was observed, from 11 ng/mL to 626.98 μg/mL, as well as high tolerability at a high dosage for a prolonged period, from 6 months to a year [78].

The first-generation type is based on the inhibition of the curcumin metabolism to enhance its absorption time by using specific adjuvants such as piperine, turmeric or fish oils and starch. The adjuvants have the role to act at the gastrointestinal level to avoid the curcumin efflux and promote the inhibition of glucoronidation at the intestinal and hepatic levels [83,84].

The second-generation formulations are characterized by the main goal to enhance the solubility of curcumin by the employment of a series of emulsifiers, including carbohydrate complexes, phospholipid complexes and polyethoxylated hydrogenated castor oil.

Finally, the third-generation formulations are focused on the administration of curcumin by using non-covalent complexes to promote the bioavailability of the compound in free form without adding synthetic emulsifiers [78,85,86].

An interesting approach for curcuminoid-based formulations for the oral administration was recently proposed according to the Polar Non-polar Sandwich (PNS) technology. The solubility and bioavailability of curcumin are promoted by combining highly polar and non-polar entities, both extracted from turmeric, to generate a natural complex matrix. The polar entities are represented by polysaccharides, proteins and fibers, while the non-polar ones consist of essential oils. The simple diffusion through the cell membrane is allowed to curcumin as a part of the non-polar sandwiched matrix, while proteins and carbohydrates generate the polar matrix. Benefits of the inflammatory diseases involved in the rheumatoid arthritis were demonstrated; moreover, antioxidant and anti-aging effects were observed by using curcuminoids for PNS technology [87,88,89,90].

To the best of our knowledge, no data are yet available in the case of cyclocurcumin-based formulations; however, promising results were recently described in the formation of the host/guest inclusion complex sulfobutylether-β-cyclodextrin/cyclocurcumin in aqueous solution (SBE-βCD/CyCur) [33]. The SBE-βCD molecule is one of the most common β-cyclodextrin derivatives in which the replacement of the hydroxyl groups of the native cyclodextrin with an average of seven negatively charged sulfobutylether groups leads to the extension of both the hydrophilic moiety and the hydrophobic cavity of the macrocycle. In addition, the use of SBE-βCD offers suitable properties for pharmaceutical formulations such as high solubility, low toxicity and better complexation in comparison to native β-cyclodextrine [91,92,93,94]. It was demonstrated that in the SBE-βCD aqueous solution, cyclocurcumin acts as a guest by forming a supramolecular complex according to a 1:1 stoichiometry. Interestingly, the thermodynamic parameters of the SBE-βCD/CyCur interactions determined at 298 K by calorimetric analysis suggested that the complexation is a spontaneous process enthalpically driven. Moreover, the CyCur thermal *cis*-*trans* isomerization rate determined at different temperatures suggested that CyCur may interact with the SBE-βCD both in the *trans* and in the *cis* states [33].

Remarkable results to overcome the limits of curcuminoids were also observed by applying the co-crystallization technique by which non-covalent interactions take place between two or more compounds into a crystalline structure. Resorcinol and pyrogallol are generally used to generate hydrogen bonds with the curcumin molecule according to a definite stoichiometry. The data obtained for the investigated formulations revealed better solubility and hygroscopicity in comparison to curcuminoids in pure form and might promote similar studies for cyclocurcumin [31,95].

## 6. Conclusions

Cyclocurcumin represents a promising bioactive phytocompound isolated in minor content from the rhizome of *Curcuma longa* L. Structurally, CyCur is characterized by a highly hydrophobic nature with low solubility in aqueous solution and the tendency to form spherical and fluorescent aggregates with a nanometric size, as microscopically observed. Furthermore, it is sensitive to light and heat. The molecule exhibits isomerism around the ethylenic double bond by which the dominant *trans* isomer may be converted into the less stable *cis* isomer under exposition to light. The *trans*-*cis* isomerization and the irreversible thermal degradation into curcumin of the solution over time can be detected by spectroscopical techniques. Cyclocurcumin demonstrates remarkable fluorescent properties and solvatochromism according to the solvent polarity and the excitation wavelength. Computational studies demonstrated the strategic role of CyCur as an antioxidant, an enzyme inhibitor, and a neuroprotective agent. Moreover, data obtained from biological tests indicate promising activities for cardiovascular and autoimmune diseases.

Although nowadays the therapeutical effects of turmeric are still essentially attributable to curcumin due to its higher content in comparison to the other curcuminoids, the specific behavior of cyclocurcumin discussed in the studies reported in this work highlights its potentiality as a lead compound to develop molecular scaffolds involved in chemical and biological applications.

The improvement of the bioavailability is still the main challenge to be overcome for the clinical use of CyCur. The development of delivery systems based on the strategies already successfully exploited in the case of curcumin may be considered in the future.

## Figures and Tables

**Table 1:** Chemical shift assignments for *trans* and *cis* isomers of CyCur in DMSO-d6. Data from reference [36].

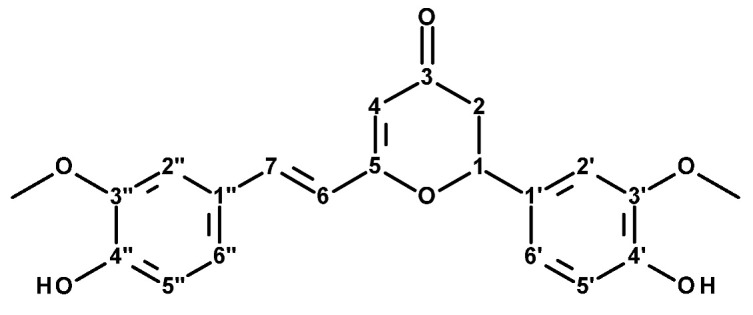	*trans* δ (ppm)	*cis* δ (ppm)
2	2.97 d, 3.02 d	2.94, 2.99
4	5.56 s	5.54 s
6	6.76 (d, J = 15.9 Hz)	5.99 (1H, d, J = 12.8 Hz)
7	7.21 (d, J = 15.9 Hz)	6.73 (1H, d, J = 12.8 Hz)
2′	7.13 d	7.05 d
5′	6.83 d	6.69 d
2″	7.24 d	6.89 d
5″	6.77 d	6.75 d
6″	7.07 dd	6.79 dd
OMe	3.81 s	3.57 s, 2.68 s

## Data Availability

Not applicable.
